# UTX/KDM6A suppresses AP-1 and a gliogenesis program during neural differentiation of human pluripotent stem cells

**DOI:** 10.1186/s13072-020-00359-3

**Published:** 2020-09-25

**Authors:** Beisi Xu, Brett Mulvey, Muneeb Salie, Xiaoyang Yang, Yurika Matsui, Anjana Nityanandam, Yiping Fan, Jamy C. Peng

**Affiliations:** 1 Center for Applied Bioinformatics, St. Jude Children’s Research Hospital, Memphis, TN 38105 USA; 2 Department of Developmental Neurobiology, St. Jude Children’s Research Hospital, Memphis, TN 38105 USA

## Abstract

**Background:**

UTX/KDM6A is known to interact and influence multiple different chromatin modifiers to promote an open chromatin environment to facilitate gene activation, but its molecular activities in developmental gene regulation remain unclear.

**Results:**

We report that in human neural stem cells, UTX binding correlates with both promotion and suppression of gene expression. These activities enable UTX to modulate neural stem cell self-renewal, promote neurogenesis, and suppress gliogenesis. In neural stem cells, UTX has a less influence over histone H3 lysine 27 and lysine 4 methylation but more predominantly affects histone H3 lysine 27 acetylation and chromatin accessibility. Furthermore, UTX suppresses components of AP-1 and, in turn, a gliogenesis program.

**Conclusions:**

Our findings revealed that UTX coordinates dualistic gene regulation to govern neural stem cell properties and neurogenesis–gliogenesis switch.

## Background

The chromatin modifier UTX/KDM6A has a crucial influence on normal development and disease. In mice, the loss of UTX leads to embryonic lethality concurrent with brain and heart malformations [1, 2]. In humans, *UTX* mutations are causally linked to developmental disorders such as Kabuki syndrome and Group 4 pediatric medulloblastoma [3–5]. Recurrent *UTX* mutations occur in 14 pediatric cancer types and 13 adult cancer types [6–9], suggesting that UTX dysfunction broadly promotes cancer progression.

UTX was originally discovered as a demethylase of histone H3-methylated-lysine 27 (H3K27me) [10–14], and has since been shown to interact with and affect the activities of H3K27 acetyltransferase P300 [15], H3K4 methyltransferases [11, 16], and the chromatin remodeler SWI/SNF [17, 18]. By removing suppressive chromatin modifications and promoting open chromatin structure, UTX presumably facilitates the activation of key developmental regulators. In mouse embryonic stem cells (ESCs), Utx mediates enhancer activation [15] and recruitment of transcription factors to chromatin [19]. Furthermore, in mice, Utx enhances the induction of pluripotency in mature fibroblasts [20]. However, the activities and influence of UTX genome wide in a developmental context remain unclear. Whether UTX facilitates gene activation only and whether it influences gene expression only through chromatin-modifying activities are also unknown. These gaps hinder the understanding of epigenetic influence over normal and diseased development as well as the etiology and intervention of diseases associated with UTX dysfunction.

We report that during differentiation of human ESCs (hESCs) into human neural stem cells (hNSCs), UTX changes most of its targets to regulate genes in transcriptional regulation, chromatin modifications, and signaling pathways. In hNSCs, UTX modulates self-renewal and coordinates neurogenesis–gliogenesis decisions by cooperating with 53BP1 [21] and suppressing AP-1 expression, subsequent AP-1-mediated chromatin accessibility, and a gliogenesis program.

## Results

### UTX can promote and suppress gene targets in hNSCs

**Fig. 1:**
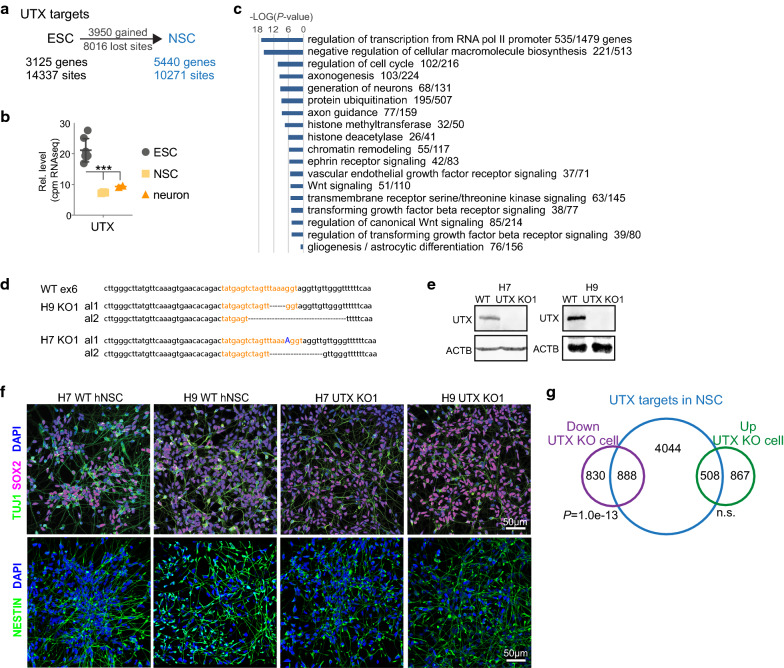
UTX binding correlates with promotion and suppression of gene expression in hNSCs. **a** UTX changes most of its target genes during the differentiation of hESCs to hNSCs. **b** Quantification of UTX transcript level in hESCs, hNSCs, and neurons. ***P < 0.001 by one-sided Student’s *t* test. **c** Gene ontology analysis of UTX-bound genes in hNSCs. Ontology terms were ranked by *P* value significance, with the number of enriched genes indicated. **d** CRISPR sgRNA sequences and mutations in UTX-KO clones. Orange sequences indicate sgRNA targets, dots indicate deletion, the blue “A” indicates an insertion, and “al” denotes allele. **e** WB analysis of UTX-WT cells and UTX-KO clones. **f** IF of neural differentiation markers in UTX-WT hESCs, UTX-WT hNSCs, and UTX-KO cells. Bar, 50 μm. **g** UTX-bound genes in hNSCs were enriched with differentially expressed genes in UTX-KO vs. UTX-WT differentiating cells. *P* values were calculated by the hypergeometric test, assuming normal data distribution

We previously used a neural differentiation course of hESCs to hNSCs and then neurons to describe the requirement of a UTX–53BP1 partnership for human neural differentiation [21]. Here, we want to more comprehensively characterize the activities of UTX during neural differentiation (Additional file 1: Figure S1A). By comparing UTX ChIP-seq datasets in hESCs and hNSCs [21], we found that UTX bound 3950 new sites but was released from 8016 sites in hNSCs compared to hESCs (Fig. 1a). We defined UTX-bound genes as those whose promoters (2 kb from transcription start sites) overlapped UTX ChIP-seq peaks (“Materials and methods”). The differential localization may be in part due to the significant downregulation of UTX expression during neural differentiation (Fig. 1b). Gene ontology (GO) analysis revealed that the UTX-bound genes in hNSC include regulators of transcription, macromolecule biosynthesis, cell cycle, generation of neurons, chromatin modifications, ephrin signaling, VEGF signaling, WNT signaling, and TGFβ signaling (Fig. 1b). In contrast, GO analysis of UTX-bound genes in hESCs include RNA-binding proteins, focal adhesion, translational regulation, nonsense-mediated decay, and ribosome assembly (Additional file 1: Figure S1B). These results suggest that UTX target genes change during neural differentiation, from regulation of RNA-binding, focal adhesion, translation, and non-sense-mediated decay in hESCs to the regulation of transcription, cell cycle, cell differentiation, chromatin structure, and signaling pathways in hNSCs.

We used the H7 and H9 hESCs to more comprehensively characterize the role of UTX during neural differentiation. We used two female hESC lines and not male hESCs (such as H1) because UTX has a functional homolog, UTY, whose gene locus locates at the Y chromosome. UTX and UTY has strong functional overlaps and the use of male lines have confounded phenotypic analyses [2, 22, 23]. We used CRISPR–Cas9 to generate UTX knockout (KO) that generated early translational stops in *UTX* (Fig. 1d, e). The H9 UTX-KO was used for the comparison with 53BP1-KO [21]. For UTX wild-type (UTX-WT) controls, we generated H7 and H9 hESCs expressing Cas9 and sgRNAs that target the *luciferase* locus and have no specificity to the human genome. UTX protein was detectable in UTX-WT hESCs but not in UTX-KO hESCs (Fig. 1e). Whole-genome sequencing of UTX-WT and UTX-KO hESCs confirmed the absence of off-target mutations (Additional file 1: Figure S1C–F).

UTX-WT and UTX-KO hESCs were differentiated along the neural lineage, and at time point ‘NSC’ of neural differentiation, they expressed known neural markers SOX2, TUJ1, NESTIN, and SOX1 (Fig. 1f, Additional file 1: Figure S2A). We used RNA-seq to profile the transcriptomes of 3 UTX-WT (H7 UTX-WT and 2 replicate H9 UTX-WT) and 4 UTX-KO (H7 UTX-KO and H9 UTX-KO1–3; Additional file 1: Figure S2B, C) hNSCs. Replicate datasets were merged in data analyses to identify consistent differences between UTX-WT and UTX-KO. We next compared UTX binding to differentially expressed genes between UTX-KO and UTX-WT hNSCs. We found that 52% (888/1718) of genes that were downregulated in UTX-KO vs. UTX-WT hNSCs were bound by UTX (*P *= 1.0e−13 by hypergeometric test assuming normal data distribution; Fig. 1g), suggesting that UTX binding in hNSCs positively correlates with their expression. Although the enrichment is not above statistical significance, 37% (508/1375) of genes with increased expression in UTX-KO vs. UTX-WT hNSCs were bound by UTX in hNSCs. These data suggest that UTX binding affects the promotion and suppression of UTX-bound genes and prompted us to investigate the effect of UTX during neural differentiation.

### UTX modulates the self-renewal property in hNSCs

**Fig. 2:**
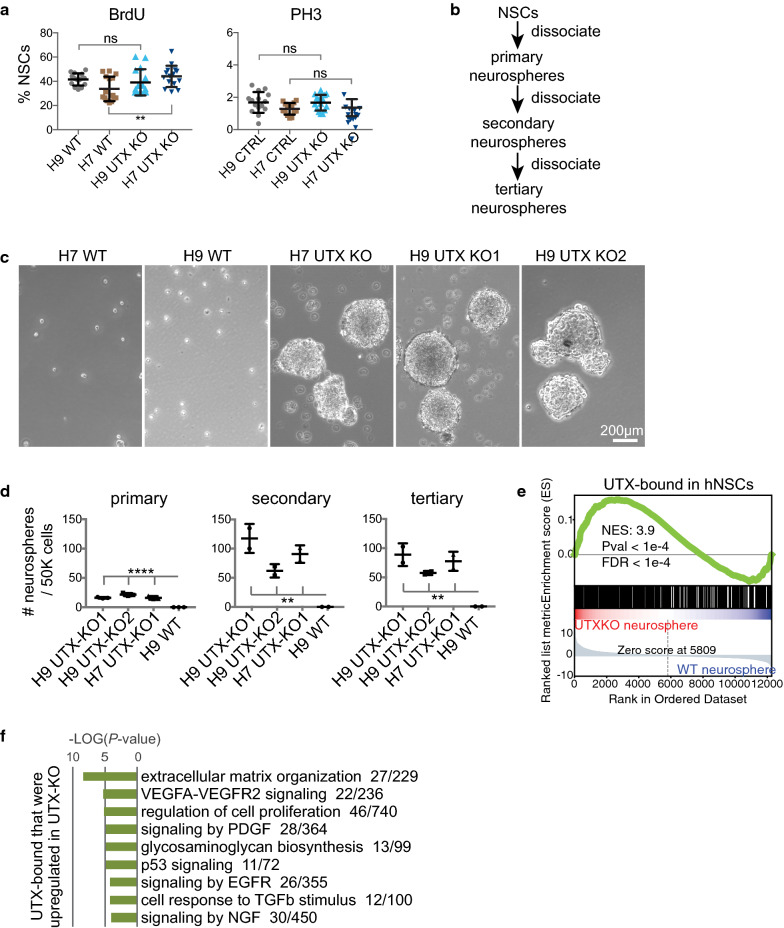
UTX-KO hNSCs are more effective than UTX-WT hNSCs at forming neurospheres. **a** Quantification of BrdU- or PH3-positive hNSCs. For each group, 15 images of more than 17,000 cells were quantified from 1 biological experiment. Each data point represents 1 image. ns, *, and ** indicate not significant, *P *< 0.05, and *P *< 0.01, respectively, by two-sided Mann–Whitney test. **b** Schematic diagram of forming and serial passaging of neurospheres. **c** Bright-field imaging of UTX-WT hNSCs and UTX-KO cells and neurospheres. **d** Quantification of neurospheres through serial passaging. **e** GSEA showed significant enrichment of UTX-bound genes in upregulated genes in UTX-KO vs. UTX-WT neurospheres. **f** Gene ontology analysis of differentially expressed genes in UTX-KO neurospheres vs. cells. Ontology terms were ranked by *P* value significance, with the number of enriched genes indicated

We examined NSC self-renewal by quantifying S phase, mitosis, and neurosphere formation. Briefly, we used the BrdU assay to detect cells in the S-phase and phospho-histone H3 (serine 10; PH3) staining to detect cells in mitosis (Additional file 1: Figure S2D). A quantitative comparison showed that more H7 UTX-KO hNSCs were in S phase than H7 UTX-WT but did not differ in PH3 quantification, and H9 UTX-KO and UTX-WT were not significantly different (Fig. 2a). To assess neurosphere formation, we serially passaged hNSCs in suspension (Fig. 2b). 53BP1 did not influence neurosphere formation (data not shown). When plated at high cell densities (50,000/6-well), UTX-WT hNSCs formed neurospheres, but UTX-KO neurospheres were densely packed and fused together. We could not accurately quantify neurospheres formed at high cell densities. When plated at low cell densities (10,000–25,000/6-well), UTX-KO hNSCs consistently formed more neurospheres than did UTX-WT across 3 passages (Fig. 2c, d), suggesting higher self-renewal of UTX-KO hNSCs.

We examined differences in gene expression between 5 UTX-KO (replicate formed by H9 UTX-KO 1–2) and 8 replicate UTX-WT neurosphere samples, which revealed 1259 downregulated genes and 1745 upregulated genes in UTX-KO neurospheres (using criteria of FDR < 0.05 and fold change > 2). Downregulated genes were highly enriched in functions related to cellular macromolecule biosynthesis, DNA metabolic processes including replication and repair, SRP-dependent cotranslational protein targeting to membrane, and mitotic phase transition. In contrast, upregulated genes were highly enriched in functions related to extracellular matrix organization, cytokine-mediated signaling, glycosaminoglycan biosynthesis, exocytosis, endoderm formation, and regulation of cell proliferation, motility, and migration (Additional file 1: Figure S2E). Glycosaminoglycans can have roles in cell signaling related to controlling proliferation and cell–cell adhesion. Gene set enrichment analysis (GSEA) showed that genes upregulated in UTX-KO neurospheres were preferentially bound by UTX in WT hNSCs (Fig. 2e). UTX-bound genes that were upregulated in UTX-KO neurospheres were enriched in functions related to extracellular matrix organization, regulation of cell proliferation, glycosaminoglycan biosynthesis, and signaling by VEGF, PDGF, p53, EGFR, TGFβ, and NGF (Fig. 2f). These processes likely influence neurosphere formation and growth. Our results suggest that in hNSCs, UTX binding leads to the suppression of genes involved in extracellular matrix organization, cell proliferation, and multiple signaling pathways, which together can influence the self-renewal property.

### UTX controls the neurogenesis vs. gliogenesis fate

To determine if UTX-KO hNSCs display a similar differentiation potential as UTX-WT hNSCs, we induced neuronal differentiation and maturation (Additional file 1: Figure S3A). UTX-KO (H7 UTX-KO and H9 UTX-KO1) and UTX-WT (H7 and H9) hNSCs cultured in neuronal differentiation media for 5 days appeared morphologically similar. However, after culture in neuronal maturation media for 6 days (the “neuron” time point in Figure S3A), UTX-WT cells condensed their nuclei and extended axons, whereas UTX-KO cells enlarged and exhibited a fibroblastic morphology (Additional file 1: Figure S3B). During neuronal differentiation, there were more H9 UTX-KO cells in the S phase than in UTX-WT, but fewer H7 UTX-KO cells in the S phase than in UTX-WT (Additional file 1: Figure S3C). Mitotic indices did not differ between the groups (Additional file 1: Figure S3C). This difference is likely caused by differences between H9 and H7 lines and not the UTX-KO mutation.

**Fig. 3:**
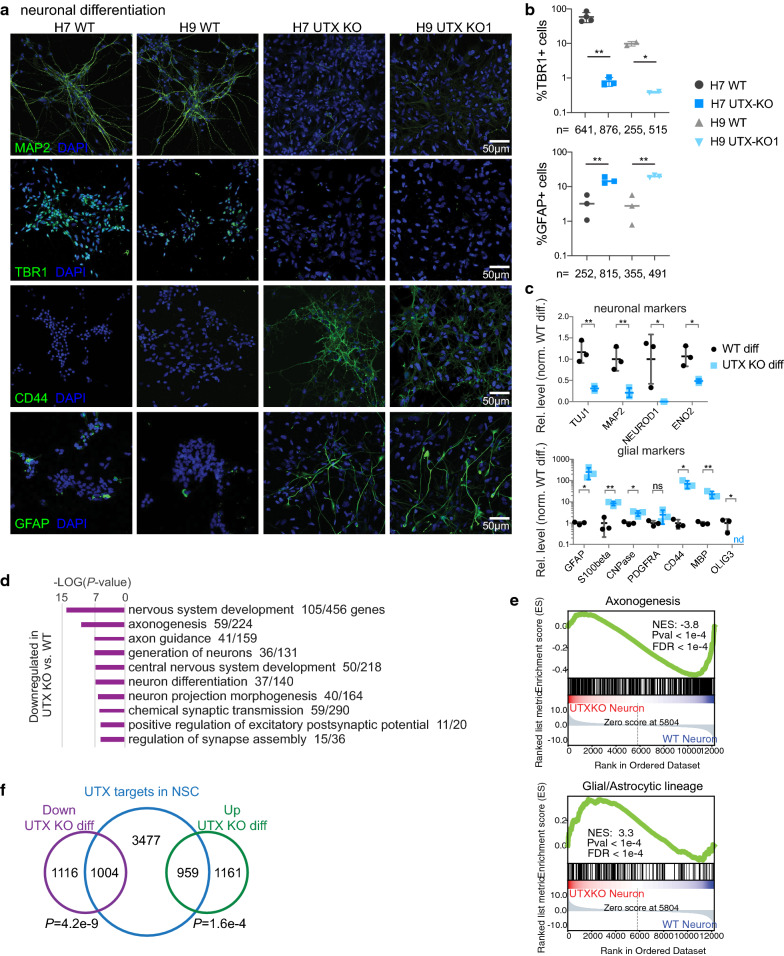
In hNSCs, UTX promotes neuronal differentiation and suppresses glial differentiation. **a** IF of neuronal (MAP2 and TBR1) and glial (CD44 and GFAP) lineage markers in UTX-WT and UTX-KO cells (H7 UTX-KO and H9 UTX-KO1) at neuron time point of differentiation. **b** Quantification of cells that are positive for the neuronal marker TBR1 or glial/astrocytic marker GFAP. More than 250 cells were sampled for each group. * and ** indicate *P *< 0.05 and 0.01. **c** Graphs summarize RNA-seq counts per million of transcripts of neuronal and glial markers in 3 H9 UTX-WT and 3 UTX-KO (H9 UTX-KO 1–2 and H7 UTX-KO) cells undergoing neuronal differentiation. **d** Gene ontology analysis of downregulated genes in UTX-KO vs. UTX-WT cells undergoing neuronal differentiation. Ontology terms were ranked by *P* value significance, with the number of enriched genes indicated. **e** GSEA showed significant enrichment of axonogenesis genes in downregulated genes and astrocytic lineage genes in upregulated genes of UTX-KO vs. UTX-WT neuronal differentiation. **g** UTX-bound genes in hNSCs were enriched with differentially expressed genes in UTX-KO vs. UTX-WT differentiating cells. *P* values were calculated by the hypergeometric test, assuming normal data distribution

The fibroblastic morphology of UTX-KO-differentiating cells resembled that of glia and, therefore, we examined the expression of neuronal and glial/astrocytic markers. After neuronal maturation, UTX-WT cells showed high expression of neuronal markers but low expression of glial/astrocytic markers. In contrast, UTX-KO cells showed high expression of glial/astrocytic differentiation markers and low expression of neuronal markers (Fig. 3a–c, Additional file 1: Figure S3D, E). RNA-seq analysis revealed numerous downregulated genes in differentiating UTX-KO (H7 UTX-KO and H9 UTX-KO 1–3) vs. UTX-WT (3 H9 replicate) cells that were enriched in functions related to nervous system development, axonogenesis, axon guidance, and generation of neurons (Fig. 3d, e), whereas upregulated genes were enriched in functions related to extracellular matrix organization, type-I interferon signaling, cytokine signaling, and neutrophil immunity (Additional file 1: Figure S3F). GSEA confirmed that downregulated genes were enriched in axonogenesis and that upregulated genes were enriched in glial/astrocytic markers (Fig. 3e). These results suggest that UTX-KO cells have decreased neuronal differentiation and increased glial/astrocytic differentiation.

To examine whether UTX gene binding in hNSCs potentially predicts gene expression patterns upon hNSC differentiation to neurons, we compared UTX-bound genes in hNSCs with genes differentially expressed in UTX-KO vs. UTX-WT cells at the “neuron” time point of differentiation. We found that 47% of genes with reduced expression in UTX-KO vs. UTX-WT “neurons” were bound by UTX in hNSCs (*P* = 4.2e−9; Fig. 3f). On the other hand, 45% of genes that were upregulated in UTX-KO vs. UTX-WT “neurons” were bound by UTX in hNSCs (*P* = 1.6e−4; Fig. 3f). These data suggest that UTX binding is strongly correlated with gene expression changes during differentiation.

Of the UTX-bound genes in hNSCs, GO analysis revealed that 68 are involved in the generation of neurons and 103 in axonogenesis (Fig. 1c). Additionally, 76 of 156 expressed genes implicated in glial/astrocyte differentiation were bound by UTX in hNSCs (Fig. 1c). Of these 76 genes, 17 known regulators of glial/astrocytic lineage were either downregulated (*CDK5R1, ID4, KMT2A, LMNB1,* and *MEGF10*) or upregulated (*ANXA7, APOE, APP, COL4A1, DRD1, EN1, FZD4, HGSNAT, HMOX1, PI4K2A, SNTA1,* and *VIM*) in UTX-KO vs. UTX-WT cells at the “neuron” time point of differentiation (Additional file 2: Table S1 lists literature information concerning the 17 genes). However, we could not unequivocally pinpoint that any of the 17 genes were crucial for UTX-KO hNSCs preferring gliogenesis at the expense of neurogenesis. These data suggest that UTX binding correlates with the positive and negative regulation of 36% (1963/5440; Additional file 1: Figure S6G) of its target genes, some of which are involved in neurogenesis and gliogenesis.

### Chromatin modification by UTX influences neurogenesis vs. gliogenesis programs

UTX is an H3K27 demethylase that physically binds to and influences the activities of H3K4 methyltransferases, H3K27 acetyltransferase P300, and the chromatin modifier SWI-SNF. We examined H3K27me3, H3K4me3, and H3K27ac by ChIP-seq and potential differential chromatin accessibility by ATAC-seq [24] at the “NSC” time point of neural differentiation, using criteria of FDR-corrected *p* < 0.05 and fold change > 2 (Additional file 1: Figure S4a–e; “Materials and methods”). For ATAC-seq analysis, we focused on nucleosome-free regions to assay significant differences between H9 UTX-KO and UTX-WT hNSCs (“Materials and methods”). UTX-bound regions showed extensive overlap with at least 1 of the 4 chromatin features (Additional file 1: Figure S4F): 7414 of 10,271 UTX-bound regions overlapped with at least one feature (*P *< 1e−15 by Fisher’s exact test), whereas only 2857 (28%) of UTX-bound regions lacked all of these features. Further analyses showed that most of these 28% of UTX-bound located within gene encoding small nucleolar RNAs or miRNAs. These findings suggest that UTX preferentially binds to chromatin regions with the assayed features in hNSCs.

To determine whether glial/astrocytic differentiation of UTX-KO is associated with changes in chromatin structure, we systematically analyzed chromatin features at promoters and distal elements 2–50 kb from the genes involved in glial/astrocytic differentiation, axonogenesis, or neuronal differentiation that are differentially expressed during neuronal differentiation. UTX-KO had little effect on H3K27me3 levels (Additional file 1: Figure S5A, B), a finding that is consistent with those of previous developmental studies showing that Utx had little to modest impact on H3K27me3 dynamics or its demethylase activity is dispensable for organismal development [1, 23, 25, 26]. Instead of H3K27me3, UTX-KO appeared to alter H3K27ac and chromatin accessibility at developmentally important genes in hNSCs. The levels of H3K27ac, H3K4me3, and chromatin accessibility were significantly altered at promoters of more than 20 developmentally important genes in UTX-KO compared with UTX-WT (Additional file 1: Figure S5C–E). UTX-KO cells also showed significantly reduced levels of H3K27ac and chromatin accessibility at distal elements of 30 developmentally important genes (Additional file 1: Figure S5C–E). Furthermore, 15 of 30 distal elements of gene loci of *TENM4*, *PAX6*, *NRXN3*, *EPHB3*, *SHANK3*, *NFASC*, *EPHB2*, *NCAM1*, *EPHB1*, *NR2E1*, *RGMA*, *CNTN4*, *ARSA*, *SMPD3*, and *FGFR2* had significantly reduced levels of both H3K27ac and chromatin accessibility (Additional file 1: Figure S5D–E), suggesting that at least 15 enhancers require UTX for proper chromatin composition. Altogether, these data suggest that UTX influences promoters and enhancers involved in neurogenesis and gliogenesis programs.

Among the features assayed, H3K27ac distribution appeared to be most disrupted by UTX-KO. We performed further analyses by segregating UTX-bound regions into two categories of significantly higher and lower H3K27ac signals in UTX-KO hNSCs. Distribution of the 2 categories among genic features and the other 3 chromatin features did not differ (Additional file 1: Figure S6A, B). However, GO analysis of genes associated with (or closest to) these regions differed. Regions with higher H3K27ac signals were enriched in functions related to miRNA-mediated inhibition of translation, Rho-guanyl-nucleotide exchange factor activity, and regulation of transcription by RNA polymerase II (Additional file 1: Figure S6C). Regions with lower H3K27ac signals were enriched with axonogenesis, axon guidance, neuron projection morphogenesis, transmembrane receptor tyrosine kinase signaling, mitogen-activated kinase, ephrin-mediated repulsion of cells, Wnt-activated receptor activity, and VEGFA–VEGFR2 signaling pathway (Additional file 1: Figure S6C). These data suggest that UTX binds and promotes H3K27ac levels at neurodevelopmentally important genes including those involved in key signaling pathways.

We next performed motif analysis to determine whether additional factors might correlate with these H3K27ac changes. Results revealed the significant enrichment of the motifs of ATOH1, TCF4, NEUROD1, MYOD, MYF5, and RRF2 in regions with lower H3K27ac signals; however, ATOH1, MYOD, and MYF5 had nondetectable expression in RNA-seq datasets (Additional file 1: Figure S6D). TCF4 and NEUROD1 both have essential role in neurogenesis [27, 28], and RFX2 is required for ciliogenesis that affects neural development [29]. There was no significant enrichment of motifs in regions with higher H3K27ac signals. We, therefore, concluded that in hNSCs, UTX-KO leads to H3K27ac increases in regions of little neurodevelopmental importance. In contrast, TCF4, NEUROD1, and RFX2 likely cooperate with or influence UTX to promote H3K27ac signals at genes involved in axonogenesis, axon guidance, neuron projection, and signaling pathways.

### UTX suppresses an AP-1-mediated program of gliogenesis

**Fig. 4:**
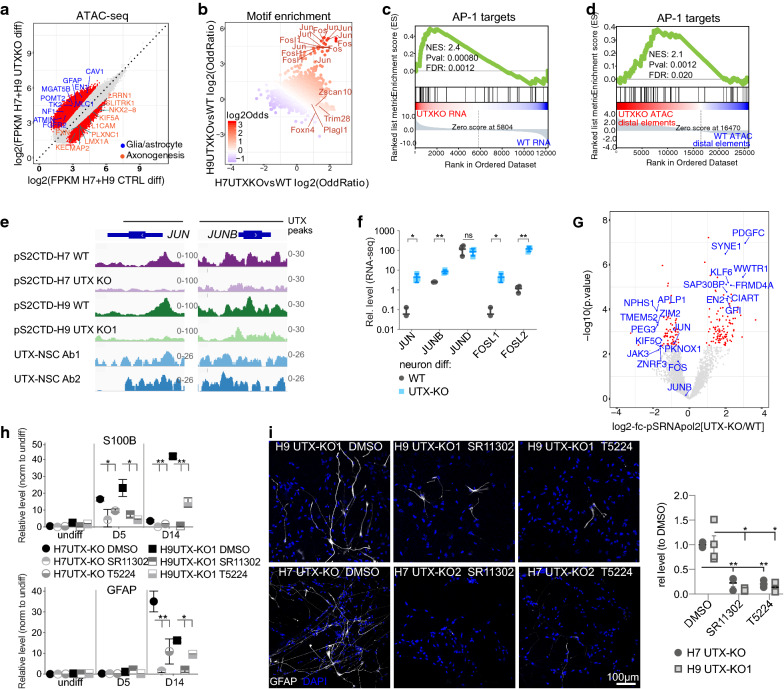
UTX suppresses AP-1 components and gliogenesis. **a** ATAC-seq datasets from H7 and H9 UTX-WT-differentiating cells were compared to those of H7 and H9 UTX-KO-differentiating cells. **b** Enrichment of consensus motifs in nucleosome-free regions (from ATAC-seq datasets) that significantly increased signals in UTX-KO vs. UTX-WT differentiating cells. GSEA showed significant enrichment of AP-1 target genes in **c** upregulated genes and **d** in distal elements with increased ATAC-seq signals in UTX-KO (vs. WT) cell differentiation. **e** At the *JUN* and *JUNB* loci, UTX ChIP-seq profiles in hNSCs and pS2-RNApol2 CUT&RUN-seq in wild-type and UTX-KO-differentiating cells. **f** Relative levels of *JUN* and *FOS* genes (normalized by counts per million from RNA-seq datasets) in UTX-WT and UTX-KO cells at neuron time point of differentiation. **g** Volcano plot of pS2-RNApol2 CUT&RUN-seq signals in UTX-KO/WT differentiating cells. fc, fold change. **h** Analysis of the transcripts of glial/astrocytic markers *S100B* and *GFAP* expressed by UTX-KO cells that were treated by DMSO or AP-1 inhibitors during neural differentiation. Observations were made with 3 biological replicates. We used 15 μM of SR-11302 and 30 μM of T-5224 at concentrations similar to those used by previous studies [48–50]. **i** GFAP immunofluorescence of UTX-KO differentiating cells that were treated by DMSO or AP-1 inhibitors. Bar, 100 μm. Quantification of GFAP-positive cells in the 6 assayed groups. More than 250 cells were counted. ns indicates not significant. * and ** indicate *P *< 0.05 and 0.01 by two-side Student’s t test

Next, we performed ATAC-seq to examine differential nucleosome-free regions in UTX-KO and UTX-WT cells at the “neuron” time point of neural differentiation, using criteria of FDR-corrected *P* < 0.05 and fold change > 2 (Fig. 4a and Additional file 1: S7A–B; “Materials and methods”). We found significantly higher ATAC-seq signals in nucleosome-free regions of H7 UTX-KO vs. WT and H9 UTX-KO vs. WT differentiating cells. These regions were enriched for consensus binding motifs of JUN and FOS proteins (Fig. 4b and Additional file 1: S7C, D), which comprise the AP-1 transcription factor complex [30]. We used published AP-1 target genes—which were downregulated by Fos shRNA and having a nearby FOS ChIP-seq peak in mouse cortical neurons [31]—for GSEA to show their significant enrichment in upregulated genes and the nucleosome-free regions of UTX-KO vs. UTX-WT differentiating cells (Fig. 4C, D). Further, promoters of multiple glial/astrocytic genes such as *CAV1*, *FGFR1*, *GFAP*, *KCNQ3*, *LTN1*, *POMT2*, *TK2*, and *VAC14* contained AP-1 motifs. Distal elements of 41 glial/astrocytic genes having significantly higher ATAC-seq signals were also AP-1 targets (Fig. 4d). However, only 19 AP-1 targets overlapped with UTX-bound genes in hNSCs. These data suggest that UTX suppresses the expression of AP-1 during neural differentiation.

Intriguingly, we found that UTX bound to *JUN* and *JUNB* loci in hNSCs (Fig. 4e), but *FOSL1 and FOSL2* loci were not bound. Moreover, *JUN, JUNB, FOSL1, and FOSL2* were upregulated in UTX-KO vs. UTX-WT cells at the “neuron” time point of differentiation (Fig. 4f and Additional file 1: S7E). The expressions of *FOS* and *FOSB* were undetectable. Potential differential chromatin accessibility, determined by ATAC-seq, at *JUN* and *JUNB* loci did not significantly differ between UTX-WT- and UTX-KO-differentiating cells, suggesting that UTX did not influence chromatin structure to suppress JUN and JUNB. Therefore, we investigated the control of RNA polymerase II in pausing–elongation, which has been proposed by others [9, 14, 32] as an alternative mechanism by which UTX regulates gene expression. We performed CUT&RUN-seq [33] (with spike-in controls; “Materials and methods”) to profile the distribution of RNA polymerase II with phosphorylated serine 2 in CTD (pS2-RNApol2) in UTX-WT and UTX-KO differentiating neurons. Compared to UTX-WT, levels of pS2-RNApol2 were lower at the promoters of *JUN* and *JUNB* loci in UTX-KO (Fig. 4e), suggesting the release of transcriptional pausing at these loci. Genome wide, pS2-RNApol2 CUT&RUN-seq signals significantly increased at 120 promoters and decreased at 99 promoters in UTX-KO vs. UTX-WT (Fig. 4g), suggesting UTX positively and negatively regulates RNA polymerase II pausing–elongation at some genes. Although the reduction of pS2-RNApol2 signal at *JUNB* locus did not pass the statistical threshold, the distribution was markedly disrupted by UTX-KO (Fig. 4e), suggesting that UTX may affect distribution as well as the total level of pS2-RNApol2 at potentially more genes.

We performed motif analysis to determine whether additional factors might correlate with differential gene expression in UTX-KO hNSCs. This analysis found significant enrichment of motifs of AP-1 components, p73, and Bach2 in upregulated genes (Additional file 1: Figure S7F) and Sox2, REST-NRSF, Sox4, Sox17, and Sox15 in downregulated genes of UTX-KO (Figure S7G). *JUN* and *JUNB* loci contained the Jun-AP1 motif. Our data suggest a network of transcription factors including AP-1 cooperate with or influence UTX to positively or negatively regulate gene expression in hNSCs.

To test whether aberrant AP-1 activity influences gene expression in UTX-KO differentiating cells, we employed two specific inhibitors of AP-1, SR-11302 [34] and T-5224 [35] (Additional file 1: Figure S7H). Compared with DMSO control treatment, inhibitor treatments did not affect the expression of NSC and neuronal markers or cell morphology, but significantly reduced the aberrant upregulation of glial/astrocytic markers S100B and GFAP in UTX-KO differentiating cells (Fig. 4h, i and Additional file 1: FigureS7I). Thus, inhibiting AP-1 activity suppresses glial/astrocytic gene expression in UTX-KO cells. As the differentiation course and drug treatment took 15 days (Additional file 1: Figure S7H), the effects of AP-1 inhibitor on UTX-KO differentiation might not be direct. Overall, our data suggest that UTX is required for AP-1 suppression, that AP-1 influences glial/astrocytic lineage genes, and that loss of UTX triggers the upregulation of AP-1 and promotion of gliogenesis.

## Conclusions

Our findings suggest that during neural differentiation, UTX modulates stem cell self-renewal and coordinates neurogenesis–gliogenesis programs. UTX executes these influences by positively and negatively regulating UTX-bound genes. Such a dualistic gene regulation is achieved by combining H3K27 acetylation, open chromatin alteration, interaction with 53BP1, influence over pausing–elongation of RNA polymerase II, and suppression of AP-1.

After an initial amplification, NSCs enter the neurogenic phase and then switch to gliogenic phase during neural development. The H3K27 methyltransferase, PRC2, is a key regulator of the neurogenesis–gliogenesis switch [36]. Depletion of PRC2 subunits led to prolonged/increased neurogenic phase and delayed/decreased gliogenic phase [36]. Our findings suggest that in contrast to the influence of PRC2, UTX promotes neurogenesis and inhibits gliogenesis. UTX exerts this specific developmental influence by regulating hundreds of its target genes that include the suppression of AP-1 subunits and the gliogenic program. This specificity highlights the importance of UTX in executing cell type- and lineage-specific gene expression programs during human neural development.

*UTX* mutations are causally linked to neurodevelopmental disorders including Group 4 pediatric medulloblastoma [8] and the Kabuki syndrome, whose patients also have high cancer predisposition and growth delays [3, 4]. Our study identified the roles of UTX in different phases of neural differentiation. UTX likely binds genes in hNSCs to promote or suppress their expression. Mechanistically, UTX potentially interacts with crucial transcription factors to affect expression of key factors involved in multiple different signaling pathways to impact NSC self-renewal and neurogenesis–gliogenesis choices. Comparative studies of UTX and 53BP1 in human stem cells [21] and our AP-1 inhibitor treatment of UTX-KO differentiating cells suggest that UTX cooperates with 53BP1 and suppresses AP-1 to regulate gene expression and hNSC functions. Further, UTX loss led to significant chromatin alterations at promoters and distal elements of genes involved in neuronal differentiation, axonogenesis, and glial/astrocytic differentiation, suggesting that UTX affects chromatin dynamics and expression of developmentally important genes in hNSCs. Our findings potentially inform the cellular and molecular defects in the UTX-linked neurodevelopmental disorders and may advance modeling of these disorders.

## Materials and methods

### Buffers

PBS: 137 mM NaCl, 2.7 mM KCl, 10 mM Na_2_HPO_4_, 1.8 mM KH_2_PO_4_ (pH 7.4). PBST: PBS with 0.1% Triton X-100. HEPM: 25 mM HEPES (pH 6.9), 10 mM EGTA, 60 mM PIPES, 2 mM MgCl_2_. IF blocking solution: 1/3 Blocker Casein (ThermoFisher Scientific, #37528), 2/3 HEPM with 0.05% TX-100. Buffer A: 10 mM HEPES (pH 7.9), 10 mM KCl, 1.5 mM MgCl_2_, 0.34 M sucrose, 10% glycerol. Buffer B: 3 mM EDTA, 0.2 mM EGTA. Buffer D: 400 mM KCl, 20 mM HEPES, 0.2 mM EDTA, 20% glycerol. ChIP lysis buffer 3: 10 mM Tris–HCl (pH 8.0), 100 mM NaCl, 1 mM EDTA, 0.5 mM EGTA, 0.1% sodium deoxycholate, 0.5% *N*-Lauroylsarcosine. FACS antibody staining solution: PBS containing 0.5% Tween 20, 1% BSA, and 0.5 µg/µL RNase.

### Antibodies

Additional file 1: Table S2 lists all antibodies and conditions used in this study.

### Human embryonic stem cell culture and neural differentiation

H7/WA07 (WiCell) and H9/WA09 (WiCell) cells were grown on Matrigel with reduced growth factors (ThermoFisher Scientific, #354230) in mTeSR1 medium (STEMCELL Technologies, #85850) at 37 °C and 5% CO_2_. For neural differentiation, ESCs were seeded onto AggreWell800 plates (STEMCELL Technologies, #34811) and fed with neural induction medium (STEMCELL Technologies, #05835) to form uni-sized embryoid bodies (EBs). On day 5, EBs were re-plated onto Matrigel-treated six-well plates. Neural differentiation (STEMCELL Technologies, #08500) started from day 11 to day 16. On day 17, cells were treated with Accutase^®^ (STEMCELL Technologies) and seeded and fed with neuronal maturation medium (STEMCELL Technologies, #08510) till days 22–24.

### Gene editing by Cas9–CRISPR and ESC clone generation

sgRNAs were designed using the clustered regularly interspaced short palindromic repeats (CRISPR) Design Tool (http://crispr.mit.edu/) to minimize off-target effects. The KO sgRNA targeting exon 6 of *UTX* is 5′TATGAGTCTAGTTTAAAGGT3′. Oligos (Integrated DNA Technologies) were synthesized, annealed, and cloned into vector LentiCRISPRv2 (Addgene plasmid #52961). sgRNAs targeting the firefly luciferase gene (exons 6 and 7) were used as non-specific controls. Viral constructs were co-transfected with VSVL, REV, and HDL into 293T cells using Lipofectamine 3000 (Life Technologies) to generate lentiviruses. Lentiviral particles were harvested in mTeSR1, and titer was quantified by the Lenti-X RT-qPCR titration kit (Clontech). For lentiviral transduction, ESCs were inoculated two consecutive times with lentiviral particles (3 h for each time) and allowed to recover in mTeSR1 overnight. Puromycin (0.375 g/mL; ThermoFisher Scientific) selection started approximately 24 h after transduction.

### Whole-genome sequencing

To evaluate the off-target effect in cells edited by CRISPR–Cas9, genomic DNAs of wild type, control, and each UTX-KO clones were extracted and analyzed by WGS analysis, using CLC Genomics Workbench v9.0 (CLC Bio) for mapping, human genome hg19 as the reference, variant calling, and coverage summarization. Indels from each sample were first called to detect editing-specific small insertions and deletions. Indels within coding regions or altered splicing donor/acceptor sites with at least 5× coverage and 5% mutation alleles were cross-validated among samples, followed by manual confirmation to remove any indels called due to sequencing or mapping errors. All other non-functionally-related indels were screened against predicted off-target sites, up to 4-bp mismatches, according to the CRISPR–Cas9 target online predictor CCTop. To better identify editing-specific longer deletions, relative coverage among samples was summarized using the average of *p* values calculated for each of the positions in a region greater than 50 bp. A *p* value of 10^−4^ was used as a threshold for low- coverage regions. To further investigate the longer insertions missed by the above indel analysis, soft-clipped reads with more than a 10-bp overhang were extracted, soft-clipping breakpoints were summarized as genomic locations, cross-filtering between samples was performed to remove non-specific locations, and potential insertion sites were defined as a pair of bidirectional breakpoints within a 5-bp window. Manual confirmation, along with coverage filtering, was performed to remove any insertion candidates caused due to sequencing or mapping errors.

### Western blotting

Equal amounts of nuclear extracts were separated by SDS-PAGE and transferred onto a nitrocellulose membrane (Bio-Rad). Membranes were blocked with 2% bovine serum albumin (BSA) in HEPM, incubated in primary antibodies (HEPM containing 1% BSA and 0.1% Triton X-100) overnight at 4 °C, washed in PBST, incubated in IRDye^®^-conjugated secondary antibodies (LI-COR), and imaged on an Odyssey^®^ Fc imaging system (LI-COR). Signals were quantitated with the Image Studio™ software (version 1.0.14; LI-COR). Student’s *t* test was used for statistical analyses.

### Immunofluorescence

Cells cultured on microscope chamber slides (Millipore) were rinsed with PBS, fixed with 3% paraformaldehyde in PBS for 5 min, permeabilized by PBST for 1–3 h, and blocked with IF blocking solution for 2–3 h. Cells were then incubated in diluted primary antibodies in blocking solution at 4 °C overnight, washed with PBST, incubated in diluted fluorescent dye-conjugated secondary antibodies for 2 h, washed with PBST, counterstained with DAPI, and mounted in ProLong Gold antifade mountant (Life Technologies, #P36930). Images were acquired with Zeiss LSM780 or Nikon C2.

### RNA-seq

Paired-end 100-cycle sequencing was performed in HiSeq 2000 or HiSeq 4000 sequencers as per the manufacturer’s instructions (Illumina). RNA-seq was mapped as previously described to human genome hg19, and HTSeq (version 0.6.1p1) [37] was used to estimate counts per million based on GENCODE (version 24lift37). After normalization by trimmed mean of M values (TMM) normalization method, Voom [38] was used to identify differentially expressed genes. Principal component analysis was performed using R, and figures were generated by ggbiplot. Pathway analysis was performed using Enrichr [39]. For gene set enrichment analysis, gene sets were put together using the MSigDB database (C2, v6.0) [40] and then analyzed using prerank mode (version 3.0) on log2 fold change from Voom analysis. To curate the glial/astrocyte-related gene list from Mousemine [41] (2018-11-09), search terms abnormal astrocyte morphology (MP:0002182), abnormal astrocyte physiology (MP:0008916), and increased astrocytoma incidence (MP:0010277) were used (Additional file 2: Table S1).

### Chromatin immunoprecipitation sequencing

Cells were harvested in PBS. Cytoplasmic fractions were extracted using buffer A with 1× protease inhibitors and 1 mM DTT. Nuclear pellets were cross-linked by 1.1% formaldehyde in buffer B with 1× protease inhibitors and 1 mM DTT; washed; and lysed in lysis buffer 3 with 1× protease inhibitors, 1 mM DTT, and 1 mM PMSF. The fixed and lysed nuclear extract was sonicated with Bioruptor^®^ Pico (Diagenode) 10 times for 15 s each, with 45-s intervals. Chromatin was added to Dynabeads™ (Life Technologies) prebound with 4 µg of antibodies. For UTX chromatin immunoprecipitation (ChIP), chromatin and antibodies were incubated overnight, followed by 4 h of recapture with Dynabeads™ the next day. After incubation, beads were washed and immunoprecipitates were eluted. DNA from eluates was recovered by the GeneJET FFPE DNA purification kit (ThermoFisher Scientific, #K0882).

Single-end reads of 50 bp were mapped to human genome hg19(GRCh37-lite) by BWA (version 0.7.12-r1039, default parameter) [42]. Duplicated reads were marked with Picard (version 2.6.0-SNAPSHOT) [43], and only non-duplicated reads were kept by samtools (parameter “-q 1 -F 1024” version 1.2). ENCODE guidelines [44] were followed for quality control of data. For peak calling of H3K27ac and H3K4me3, MACS2 (version 2.1.1.20160309) was used [45]. Peaks for UTX were called by SICER (V1.1) [46], with parameter “1200 fragment size 0.86600 0.00001”. For Yang et al. [21], we called peaks with MACS2 (version 2.0.9 20111102) and combined MACS2-called peaks with SICER-called peaks to ensure that methodology was optimal to identify colocalization between UTX and 53BP1. To ensure replicability, reproducible peaks for each group were finalized as only peaks retained if called with a stringent cutoff (-q 0.05) in one sample and at least called with a lower cutoff (-q 0.5) in the other sample. Peaks for H3K27me3 were called by reproducible peaks between SICER (V1.1) and MACS2. Correlation plots indicated that libraries were reproducible between biological replicates. After TMM normalization, the empirical Bayes test was used after linear fitting from Voom package (R 3.23, edgeR 3.12.1, limma 3.26.9) [38] to find differential binding sites. FDR-corrected *P* value 0.05 and fold change > 2 were used as cutoff.

### ATAC-seq

Libraries were generated from isolated nuclei using a standard protocol [24]. 2× 100-bp paired-end reads obtained from all samples were trimmed for Nextera adapter by cutadapt (version 1.9, paired-end mode, default parameter with “-m 6 -O 20”) and aligned to human genome hg19 (GRCh37-lite) by BWA (version 0.7.12-r1039, default parameter). Duplicated reads were then marked with Picard (version 2.6.0-SNAPSHOT) [43], and only non-duplicated proper paired reads were kept by samtools (parameter “-q 1 -F 1804” version 1.2). After adjusting Tn5 shift (reads were offset by +4 bp and –5 bp, respectively, for sense and antisense strands), reads were separated into nucleosome free, mononucleosome, dinucleosome, or trinucleosome, as described by Buenrostro et al. [24] by fragment size and bigwig files were generated using the center 80 bp of fragments and scale to 30 M nucleosome-free reads. There were reasonable nucleosome-free peaks and pattern of mononucleosome, dinucleosome, and trinucleosome on IGV (version 2.4.13) [47], and all samples had about 10 M nucleosome-free reads, confirming that data qualities were good and had enough depth. Next, each of the two replicates were merged to enhance peak calling on nucleosome-free reads by MACS2 (version 2.1.1.20160309 default parameters with “–extsize 200–nomodel “) [45]. To ensure replicability, reproducible peaks were finalized for each group as only those retained if called with a stringent cutoff (macs2 -q 0.05) in one merged sample and at least called with a lower cutoff (macs2-q 0.5) in the other merged sample. Then reproducible peaks were further merged between groups to create a final set of reference chromatin-accessible regions. To find differentially accessible regions, normalized raw nucleosome-free reads counts were first normalized using the TMM and the empirical Bayes statistics test was applied after linear fitting from Voom package (R 3.23, edgeR 3.12.1, limma 3.26.9) [38]. FDR-corrected *p* value 0.05 and fold change > 2 were used as the cutoff for differentially accessible regions.

### Neurosphere assay

Human NSCs were treated with Accutase (STEMCELL Technologies, Cat. No. 07920), dissociated into single cells, and plated in low-attachment six-well plates (Fisher Scientific, #CLS3471) at 10,000–25,000 cells/six-well densities. For serial passages, neurospheres were cultured for 6–8 days and then dissociated into single cells with Accutase treatment and plated in neural expansion media.

### BrdU labeling and detection

Cells plated on Millicell EZ chamber slides (Millipore, cat. no. PEZGS0416) were cultured in media containing 10uM 5-BrdU (Sigma-Aldrich, #B5002) for 5 h at 37 °C. Cells were then washed with PBS and fixed with 3% paraformaldehyde in PBS for 10 min. To denature DNA, cells were incubated with 2 N HCl in PBST for 1 h, washed with PBST, permeabilized in 0.1% PBST for 1–3 h, and blocked with immunofluorescence blocking buffer for 2–3 h. To detect BrdU and PH3, cells were incubated with anti-PH3 antibody in blocking solution at 4 °C overnight, washed with PBST, incubated with Alexa Fluor^®^ 488 anti-BrdU antibody and Alexa Fluor^®^ 647 AffiniPure Donkey Anti-Mouse IgG (H + L) for 2 h, washed with PBST, stained with DAPI, and mounted in ProLong Gold antifade mountant (Life Technologies, #P36930). Images were acquired with the Keyence BZ-X700 microscope.

### CUT&RUN

We followed CUT&RUN described in Skene and Benioff [33], with minor variations. Samples and spike-in S2 cells were pelleted and resuspended in wash buffer together. Bio-Mag Plus Concanavalin-A-coated beads (Bangs Laboratories BP531) were added to cells (diluted in binding buffer) to bind nuclei to beads. Supernatant was removed and samples were blocked for 5 min at RT with digitonin block buffer. pS2-RNApol2 and spike-in *Drosophila* H2Av antibodies diluted in digitonin block buffer were added and samples were rotated for 5 h at 4 °C. Beads were collected and washed 3 × with digitonin block buffer before adding pA-MNase to beads. After a 1-h incubation, beads were washed 3× and resuspended in wash buffer. After equilibration on ice, 100 mM CaCl_2_ was added to tubes and samples were incubated for 25 min with agitation at the 15-min mark. The reaction was stopped by adding the stop buffer. Samples were incubated at 37 °C for 30 min to release chromatin. DNA was isolated by phenol–chloroform–isoamyl alcohol extraction and MaXtract phase-lock tubes (QIAGEN) to maximally retain chromatin. Chromatin was resuspended in low-EDTA TE buffer and analyzed by TapeStation using the HS DNA Kit (Agilent). Libraries were made using the Accel-NGS^®^ 1S Plus DNA Library Kit (Swift Biosciences) and submitted for 50 bp paired-end sequencing. In all cases, ~ 15% input was removed and IgG was used as a negative control. Analysis pipeline was the same as ChIP-seq. Normalized (to *Drosophila* reads) pS2-RNApol2 CUT&RUN-seq reads at promoters (2 kb within transcription start sites) were analyzed.

## Supplementary information

**Additional file 1.** Supplementary Figures S1–S7 and supplementary Table S1.

**Additional file 2.** List of citations supporting UTX-bound genes involved in glial/astrocytic differentiation.

## Data Availability

All sequencing data are deposited in GEO: GSE127515 & GSE108116.

## References

[1] Wang C, Lee JE, Cho YW, Xiao Y, Jin Q, Liu C, Ge K (2012). UTX regulates mesoderm differentiation of embryonic stem cells independent of H3K27 demethylase activity. Proc Natl Acad Sci USA.

[2] Welstead GG, Creyghton MP, Bilodeau S, Cheng AW, Markoulaki S, Young RA, Jaenisch R (2012). X-linked H3K27me3 demethylase Utx is required for embryonic development in a sex-specific manner. Proc Natl Acad Sci USA.

[3] Cheon CK, Sohn YB, Ko JM, Lee YJ, Song JS, Moon JW, Yang BK, Ha IS, Bae EJ, Jin HS (2014). Identification of KMT2D and KDM6A mutations by exome sequencing in Korean patients with Kabuki syndrome. J Hum Genet.

[4] Miyake N, Mizuno S, Okamoto N, Ohashi H, Shiina M, Ogata K, Tsurusaki Y, Nakashima M, Saitsu H, Niikawa N (2013). KDM6A point mutations cause Kabuki syndrome. Hum Mutat.

[5] Skowron P, Ramaswamy V, Taylor MD (2015). Genetic and molecular alterations across medulloblastoma subgroups. J Mol Med.

[6] van Haaften G, Dalgliesh GL, Davies H, Chen L, Bignell G, Greenman C, Edkins S, Hardy C, O’Meara S, Teague J (2009). Somatic mutations of the histone H3K27 demethylase gene UTX in human cancer. Nat Genet.

[7] Van der Meulen J, Speleman F, Van Vlierberghe P (2014). The H3K27me3 demethylase UTX in normal development and disease. Epigenetics.

[8] Zhou X, Edmonson MN, Wilkinson MR, Patel A, Wu G, Liu Y, Li Y, Zhang Z, Rusch MC, Parker M (2016). Exploring genomic alteration in pediatric cancer using ProteinPaint. Nat Genet.

[9] Wang L, Shilatifard A (2019). UTX mutations in human cancer. Cancer Cell.

[10] Agger K, Cloos PA, Christensen J, Pasini D, Rose S, Rappsilber J, Issaeva I, Canaani E, Salcini AE, Helin K (2007). UTX and JMJD3 are histone H3K27 demethylases involved in HOX gene regulation and development. Nature.

[11] Hong S, Cho YW, Yu LR, Yu H, Veenstra TD, Ge K (2007). Identification of JmjC domain-containing UTX and JMJD3 as histone H3 lysine 27 demethylases. Proc Natl Acad Sci USA.

[12] Lan F, Bayliss PE, Rinn JL, Whetstine JR, Wang JK, Chen S, Iwase S, Alpatov R, Issaeva I, Canaani E (2007). A histone H3 lysine 27 demethylase regulates animal posterior development. Nature.

[13] Lee MG, Villa R, Trojer P, Norman J, Yan KP, Reinberg D, Di Croce L, Shiekhattar R (2007). Demethylation of H3K27 regulates polycomb recruitment and H2A ubiquitination. Science.

[14] Smith ER, Lee MG, Winter B, Droz NM, Eissenberg JC, Shiekhattar R, Shilatifard A (2008). Drosophila UTX is a histone H3 Lys27 demethylase that colocalizes with the elongating form of RNA polymerase II. Mol Cell Biol.

[15] Wang SP, Tang Z, Chen CW, Shimada M, Koche RP, Wang LH, Nakadai T, Chramiec A, Krivtsov AV, Armstrong SA (2017). A UTX-MLL4-p300 transcriptional regulatory network coordinately shapes active enhancer landscapes for eliciting transcription. Mol Cell.

[16] Guo C, Chang CC, Wortham M, Chen LH, Kernagis DN, Qin X, Cho YW, Chi JT, Grant GA, McLendon RE (2012). Global identification of MLL2-targeted loci reveals MLL2’s role in diverse signaling pathways. Proc Natl Acad Sci USA.

[17] Tie F, Banerjee R, Conrad PA, Scacheri PC, Harte PJ (2012). Histone demethylase UTX and chromatin remodeler BRM bind directly to CBP and modulate acetylation of histone H3 lysine 27. Mol Cell Biol.

[18] Miller SA, Mohn SE, Weinmann AS (2010). Jmjd3 and UTX play a demethylase-independent role in chromatin remodeling to regulate T-box family member-dependent gene expression. Mol Cell.

[19] Petruk S, Cai J, Sussman R, Sun G, Kovermann SK, Mariani SA, Calabretta B, McMahon SB, Brock HW, Iacovitti L (2017). Delayed accumulation of H3K27me3 on nascent DNA is essential for recruitment of transcription factors at early stages of stem cell differentiation. Mol Cell.

[20] Mansour AA, Gafni O, Weinberger L, Zviran A, Ayyash M, Rais Y, Krupalnik V, Zerbib M, Amann-Zalcenstein D, Maza I (2012). The H3K27 demethylase Utx regulates somatic and germ cell epigenetic reprogramming. Nature.

[21] Yang X, Xu B, Mulvey B, Evans M, Jordan S, Wang YD, Pagala V, Peng J, Fan Y, Patel A (2019). Differentiation of human pluripotent stem cells into neurons or cortical organoids requires transcriptional co-regulation by UTX and 53BP1. Nat Neurosci.

[22] Wang J, Sun Q, Morita Y, Jiang H, Gross A, Lechel A, Hildner K, Guachalla LM, Gompf A, Hartmann D (2012). A differentiation checkpoint limits hematopoietic stem cell self-renewal in response to DNA damage. Cell.

[23] Shpargel KB, Sengoku T, Yokoyama S, Magnuson T (2012). UTX and UTY demonstrate histone demethylase-independent function in mouse embryonic development. PLoS Genet.

[24] Buenrostro JD, Giresi PG, Zaba LC, Chang HY, Greenleaf WJ (2013). Transposition of native chromatin for fast and sensitive epigenomic profiling of open chromatin, DNA-binding proteins and nucleosome position. Nat Methods.

[25] Shpargel KB, Starmer J, Wang C, Ge K, Magnuson T (2017). UTX-guided neural crest function underlies craniofacial features of Kabuki syndrome. Proc Natl Acad Sci USA.

[26] Vandamme J, Lettier G, Sidoli S, Di Schiavi E, Norregaard Jensen O, Salcini AE (2012). The C elegans H3K27 demethylase UTX-1 is essential for normal development, independent of its enzymatic activity. PLoS Genet.

[27] Gao Z, Ure K, Ables JL, Lagace DC, Nave KA, Goebbels S, Eisch AJ, Hsieh J (2009). Neurod1 is essential for the survival and maturation of adult-born neurons. Nat Neurosci.

[28] Schmidt-Edelkraut U, Daniel G, Hoffmann A, Spengler D (2014). Zac1 regulates cell cycle arrest in neuronal progenitors via Tcf4. Mol Cell Biol.

[29] Chung MI, Peyrot SM, LeBoeuf S, Park TJ, McGary KL, Marcotte EM, Wallingford JB (2012). RFX2 is broadly required for ciliogenesis during vertebrate development. Dev Biol.

[30] Jochum W, Passegue E, Wagner EF (2001). AP-1 in mouse development and tumorigenesis. Oncogene.

[31] Malik AN, Vierbuchen T, Hemberg M, Rubin AA, Ling E, Couch CH, Stroud H, Spiegel I, Farh KK, Harmin DA (2014). Genome-wide identification and characterization of functional neuronal activity-dependent enhancers. Nat Neurosci.

[32] Wang AH, Zare H, Mousavi K, Wang C, Moravec CE, Sirotkin HI, Ge K, Gutierrez-Cruz G, Sartorelli V (2013). The histone chaperone Spt6 coordinates histone H3K27 demethylation and myogenesis. EMBO J.

[33] Skene PJ, Henikoff S (2017). An efficient targeted nuclease strategy for high-resolution mapping of DNA binding sites. Elife.

[34] Fanjul A, Dawson MI, Hobbs PD, Jong L, Cameron JF, Harlev E, Graupner G, Lu XP, Pfahl M (1994). A new class of retinoids with selective inhibition of AP-1 inhibits proliferation. Nature.

[35] Aikawa Y, Morimoto K, Yamamoto T, Chaki H, Hashiramoto A, Narita H, Hirono S, Shiozawa S (2008). Treatment of arthritis with a selective inhibitor of c-Fos/activator protein-1. Nat Biotechnol.

[36] Hirabayashi Y, Suzki N, Tsuboi M, Endo TA, Toyoda T, Shinga J, Koseki H, Vidal M, Gotoh Y (2009). Polycomb limits the neurogenic competence of neural precursor cells to promote astrogenic fate transition. Neuron.

[37] Anders S, Pyl PT, Huber W (2015). HTSeq—a Python framework to work with high-throughput sequencing data. Bioinformatics.

[38] Law CW, Chen Y, Shi W, Smyth GK (2014). voom: precision weights unlock linear model analysis tools for RNA-seq read counts. Genome Biol.

[39] Kuleshov MV, Jones MR, Rouillard AD, Fernandez NF, Duan Q, Wang Z, Koplev S, Jenkins SL, Jagodnik KM, Lachmann A (2016). Enrichr: a comprehensive gene set enrichment analysis web server 2016 update. Nucleic Acids Res.

[40] Liberzon A, Subramanian A, Pinchback R, Thorvaldsdóttir H, Tamayo P, Mesirov JP (2011). Molecular signatures database (MSigDB) 30. Bioinformatics.

[41] Motenko H, Neuhauser SB, O’Keefe M, Richardson JE (2015). MouseMine: a new data warehouse for MGI. Mamm Genome.

[42] McKenna A, Hanna M, Banks E, Sivachenko A, Cibulskis K, Kernytsky A, Garimella K, Altshuler D, Gabriel S, Daly M (2010). The genome analysis Toolkit: a MapReduce framework for analyzing next-generation DNA sequencing data. Genome Res.

[43] Li H, Handsaker B, Wysoker A, Fennell T, Ruan J, Homer N, Marth G, Abecasis G, Durbin R (2009). The sequence alignment/map format and SAMtools. Bioinformatics.

[44] Landt SG, Marinov GK, Kundaje A, Kheradpour P, Pauli F, Batzoglou S, Bernstein BE, Bickel P, Brown JB, Cayting P (2012). ChIP-seq guidelines and practices of the ENCODE and modENCODE consortia. Genome Res.

[45] Zhang Y, Liu T, Meyer CA, Eeckhoute J, Johnson DS, Bernstein BE, Nusbaum C, Myers RM, Brown M, Li W (2008). Model-based Analysis of ChIP-Seq (MACS). Genome Biol.

[46] Zang C, Schones DE, Zeng C, Cui K, Zhao K, Peng W (2009). A clustering approach for identification of enriched domains from histone modification ChIP-Seq data. Bioinformatics.

[47] Robinson JT, Thorvaldsdóttir H, Winckler W, Guttman M, Lander ES, Getz G, Mesirov JP (2011). Integrative genomics viewer. Nat Biotechnol.

[48] Yang Y, Huycke MM, Herman TS, Wang X (2016). Glutathione S-transferase alpha 4 induction by activator protein 1 in colorectal cancer. Oncogene.

[49] Chen H, Qu J, Huang X, Kurundkar A, Zhu L, Yang N, Venado A, Ding Q, Liu G, Antony VB (2016). Mechanosensing by the alpha6-integrin confers an invasive fibroblast phenotype and mediates lung fibrosis. Nat Commun.

[50] Johannessen L, Sundberg TB, O’Connell DJ, Kolde R, Berstler J, Billings KJ, Khor B, Seashore-Ludlow B, Fassl A, Russell CN (2017). Small-molecule studies identify CDK8 as a regulator of IL-10 in myeloid cells. Nat Chem Biol.

