# Supplementary material for: UTX/KDM6A suppresses AP-1 and a gliogenesis program during neural differentiation of human pluripotent stem cells
Source: Epigenetics Chromatin. 2020 Sep 25;13:38. doi: 10.1186/s13072-020-00359-3 (PMC7519529; doi:10.1186/s13072-020-00359-3)
Supplement: Supplementary file 1 — Additional file 1. Supplementary Figures S1–S7 and supplementary Table S1. [file 13072_2020_359_MOESM1_ESM.pdf]

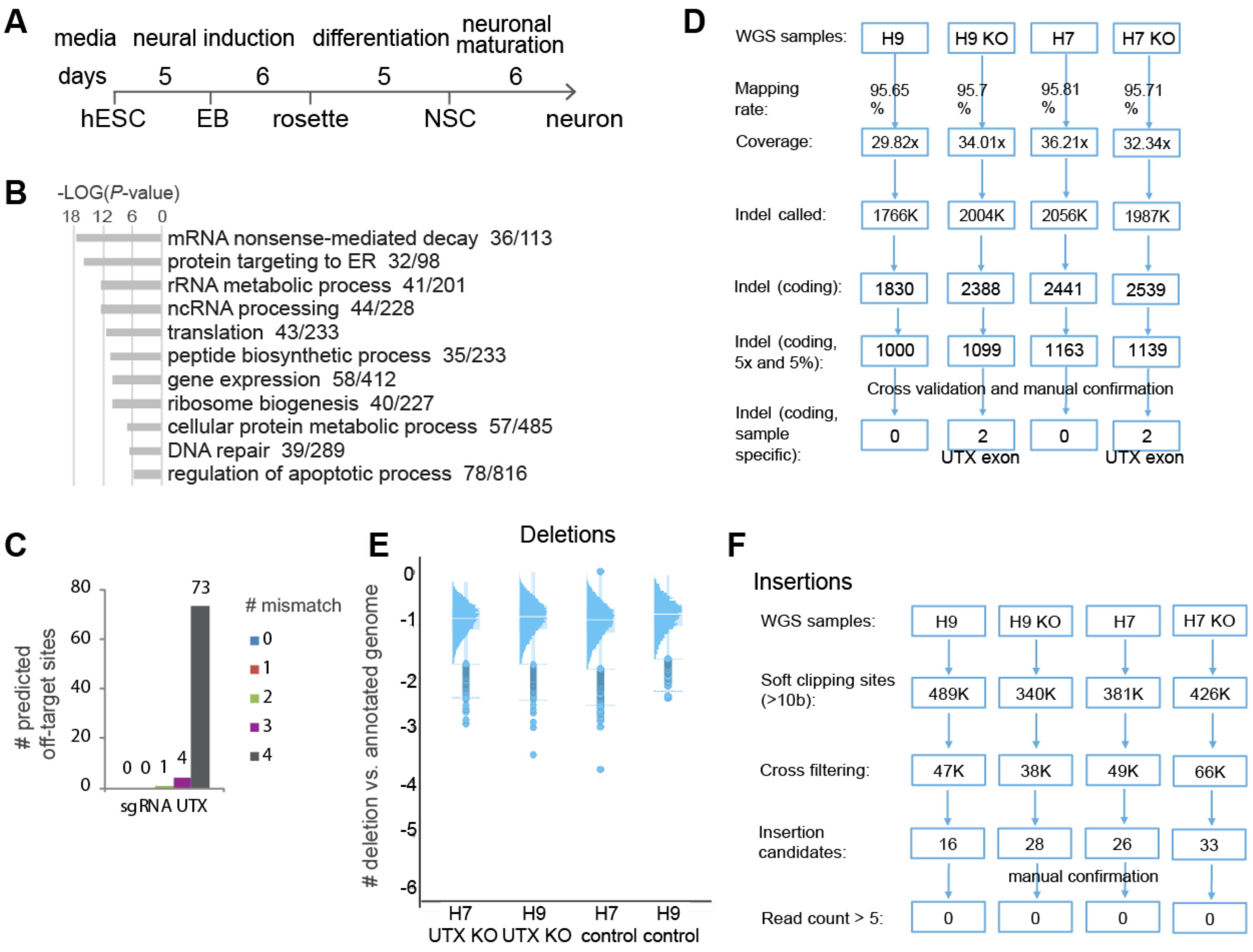

Figure S1. Whole-genome sequencing analyses and analyses of control and UTX-KO hESCs.
(A) Schematic diagram of differentiating hESCs along the neural lineage to mature neurons. Human ESC cells (Day 0) were plated in media for neural induction at D1 and then plated to form rosettes during D5-11. Rosettes were plated at D11 in neural differentiation media to form hNSCs, which were differentiated into neurons by plating in neuronal maturation media at D17. Analysis of hNSCs were done at timepoint ‘NSC’. (B) Gene ontology analysis of UTX-bound genes in hESCs. Ontology terms were ranked by *p*-value significance, with the number of enriched genes indicated. (C) Numbers of predicted off-target sites with 0-4 mismatches to the CRISPR gRNA. (D) Summary flow chart of sequencing analysis to detect small insertions and deletions (indel). Against the human reference genome (hg19), detected indels were filtered by the listed criteria, removed while present in more than one sample or sample group, and manually confirmed excluding sequencing or mapping errors. Indels specific to the UTX exon 6 were the only functionally related indel detected. (E) Deletion analysis. The relative coverage analysis among this group of samples, targeting for deletions larger than 50 bp that differed from the annotated genome. No significant difference between UTX-WT and UTX-KO samples was detected. (F) Summary flow chart of insertion analysis. The soft-clipped reads (>10 bp overhang) were extracted and used to detect larger insertions that were failed to report in previous indel analysis. Soft-clipping break points were summarized as genomic locations, cross filtering between samples was to remove non-specific locations, and the potential insertion sites were defined as a pair of bi-direction break points within a 5-bp window. No insertion was identified.

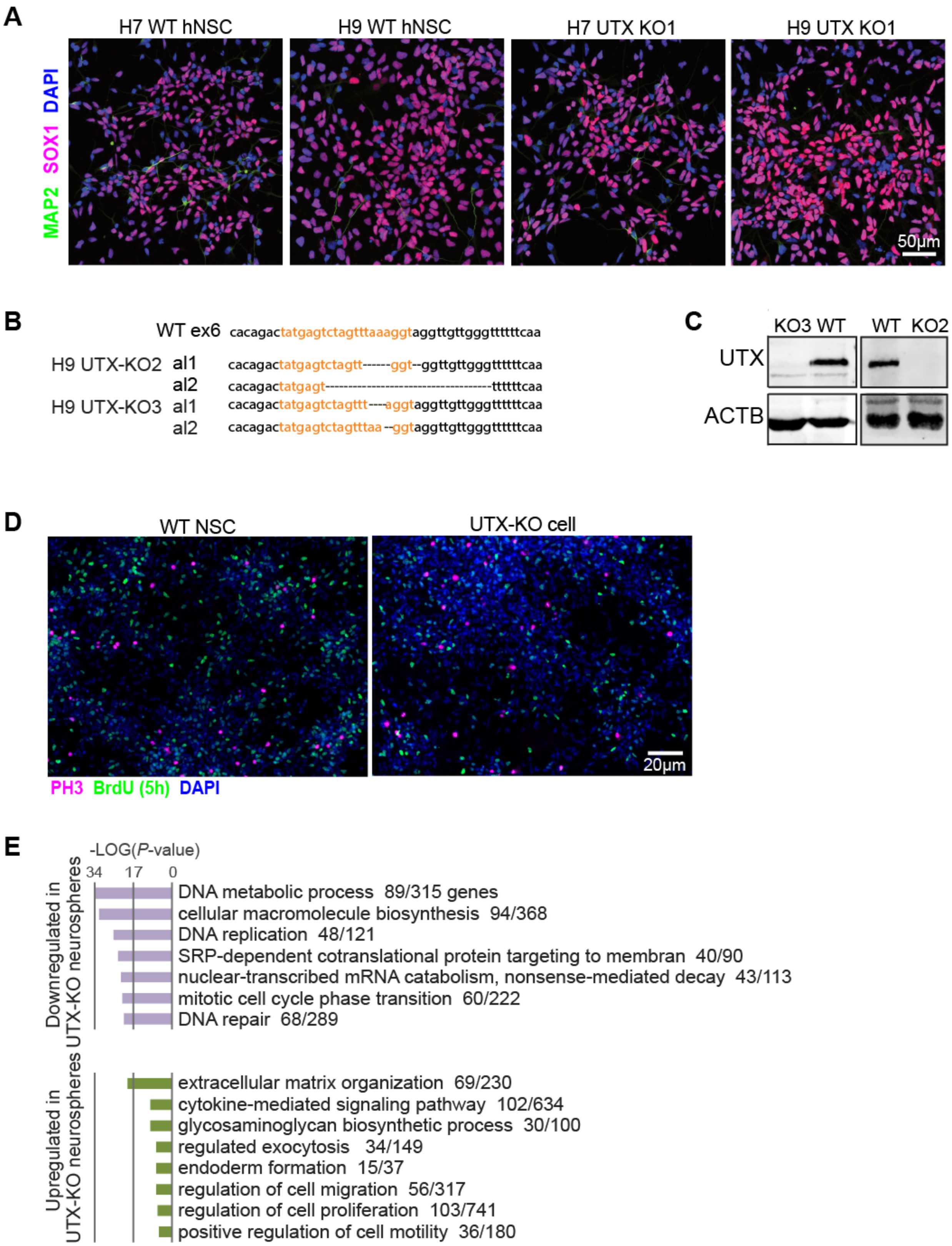

Figure S2. Additional UTX-KO clones and NSC self-renewal assay.
(A) IF of neural differentiation markers in UTX-WT and UTX-KO hNSCs. Bar, 50 μm. (B) CRISPR sgRNA sequences and mutations in UTX-KO clones. Orange sequences indicate sgRNA targets, dots indicate deletion, the blue "A" indicates an insertion, and "al" denotes allele. (C) WB analysis of UTX-WT and UTX-KO clones. (D) IF of control hNSCs and UTX-KO cells that are labeled with BrdU for 5 h. Bar, 20 μm. (E) Gene ontology analysis of differentially expressed genes in UTX-KO vs. UTX-WT neurospheres. The ontology terms were ranked by *P*-value significance, with the number of enriched genes indicated.

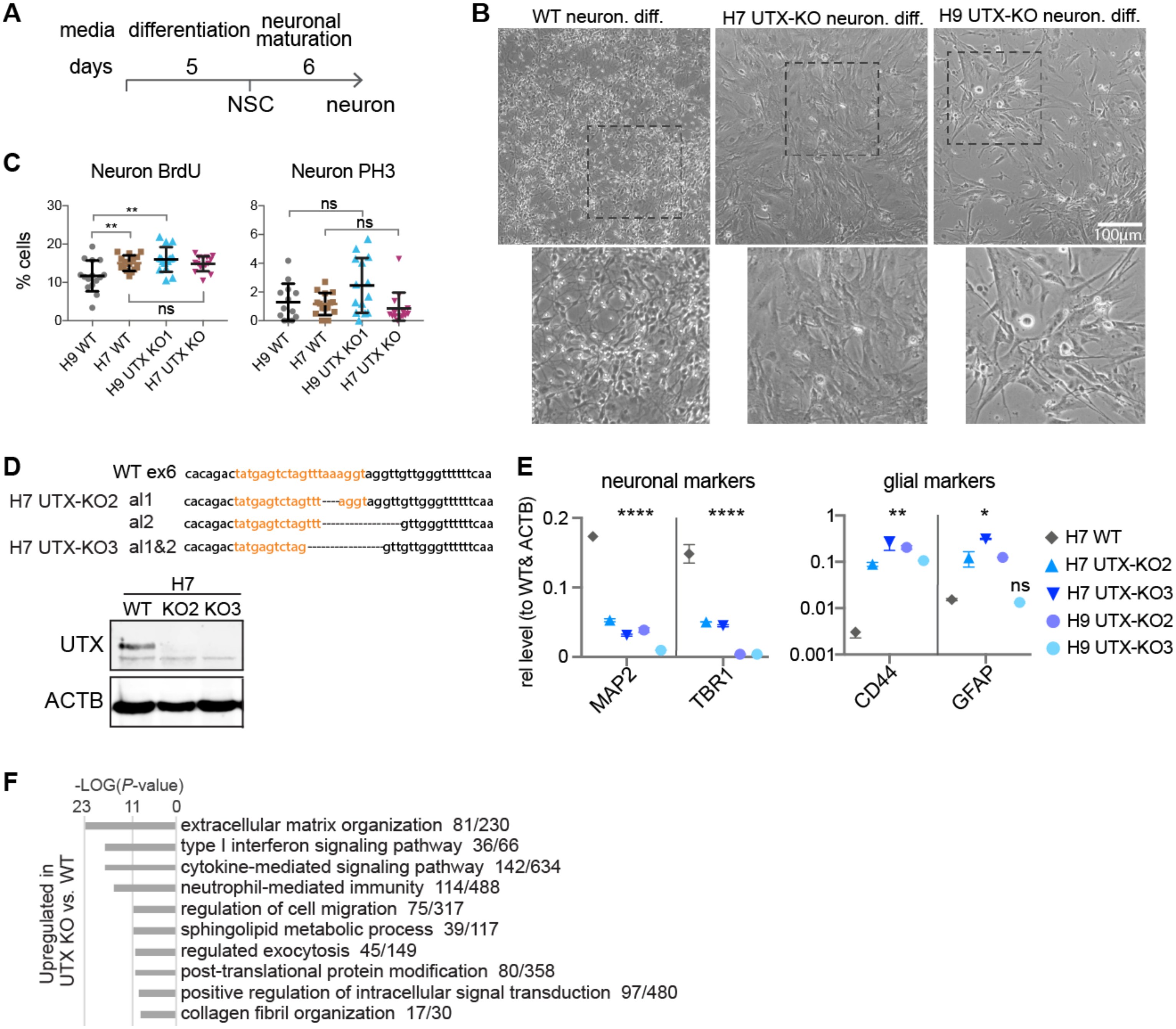

Figure S3. Neuronal differentiation of UTX-WT and UTX-KO.
(A) Diagram of hNSCs treated with neural differentiation media and then neuronal maturation media during neuronal differentiation. (B) Representative bright-field images of control and UTX-KO cells undergoing neuronal differentiation. (C) Quantification of BrdU- and PH3- positive control and UTX-KO cells undergoing neuronal differentiation. For each group, 15 images of more than 3,300 cells were quantified from 1 biological experiment. Each data point represents 1 image. ns and ** indicate not significant and $p<0.01$ respectively by 2-sided Mann–Whitney test. (D) Derivation of additional H7 UTX-KO cells. CRISPR sgRNA sequences and mutations in UTX-KO clones. Orange sequences indicate sgRNA targets, dots indicate deletion, the blue "A" indicates an insertion, and "al" denotes allele. WB analysis of UTX-WT cells and UTX-KO clones. (E) RT-qPCR analysis of neuronal markers and glial markers in UTX-WT and UTX-KO differentiating cells. (F) Gene ontology analysis of upregulated genes in UTX-KO vs. control cells undergoing neuronal differentiation. The ontology terms were ranked by *P*-value significance, with the number of enriched genes indicated.

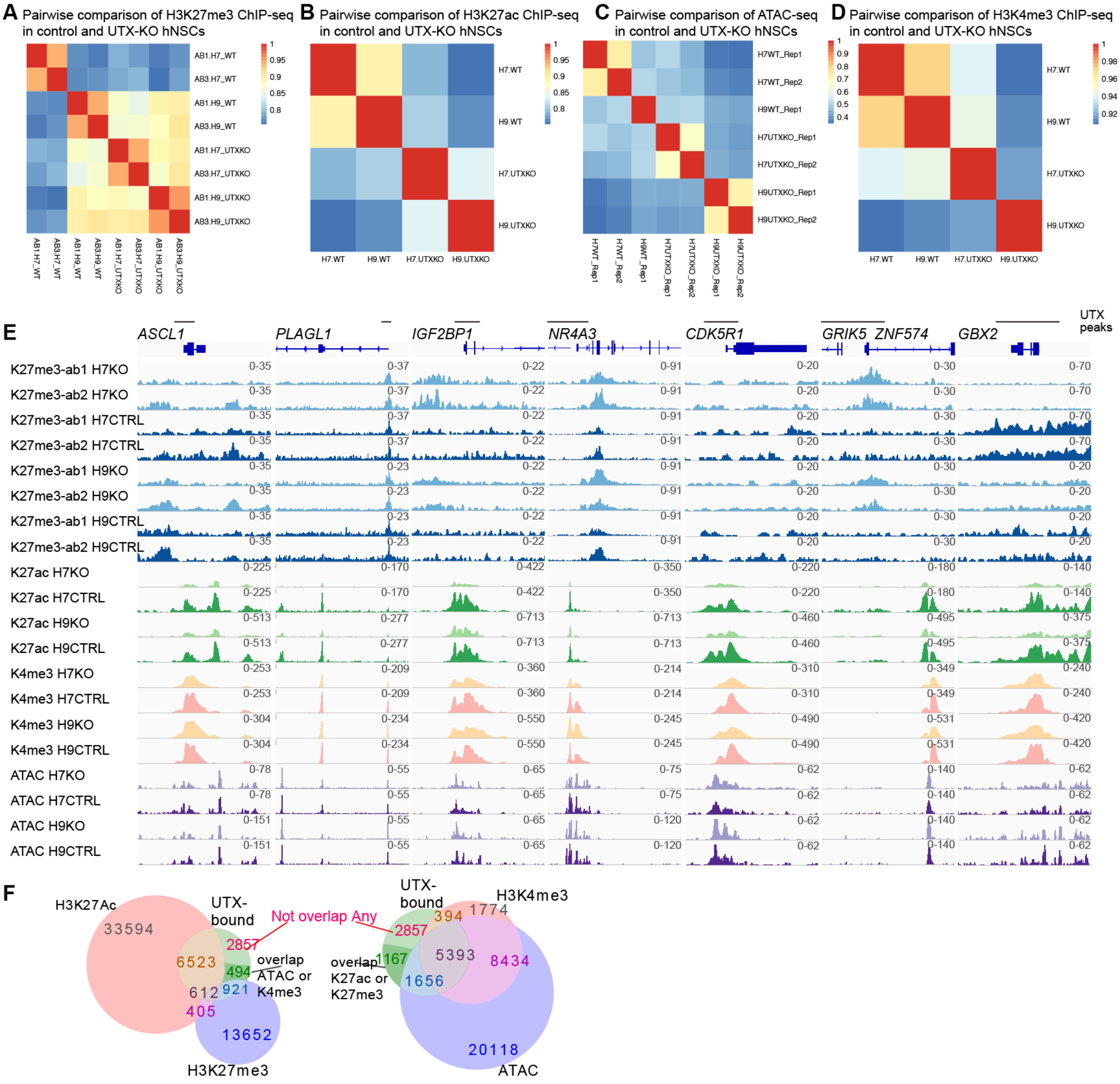

Figure S4. Comparison of chromatin features in UTX-KO cells vs. UTX-WT hNSCs.
Pairwise comparisons of datasets of (A) H3K27me3, (B) H3K27ac, (C) ATAC-seq, and (D) H3K4me3 in control and UTX-KO hNSCs. Each dataset is ChIP-seq signals at binned genomic regions. Pair-wise counts per million of the datasets with respective R^2 values indicated were shown. Red indicated higher density of points. (E) Representative profiles of histone ChIP-seq and ATAC-seq at 8 loci in H7 and H9 control and UTX-KO hNSCs. (F) Venn diagrams showing the overlap between UTX-bound regions and the regions containing the 4 assayed chromatin features. We constructed 2 Venn diagrams because we could not fit 5 lists in 1 Venn diagram.

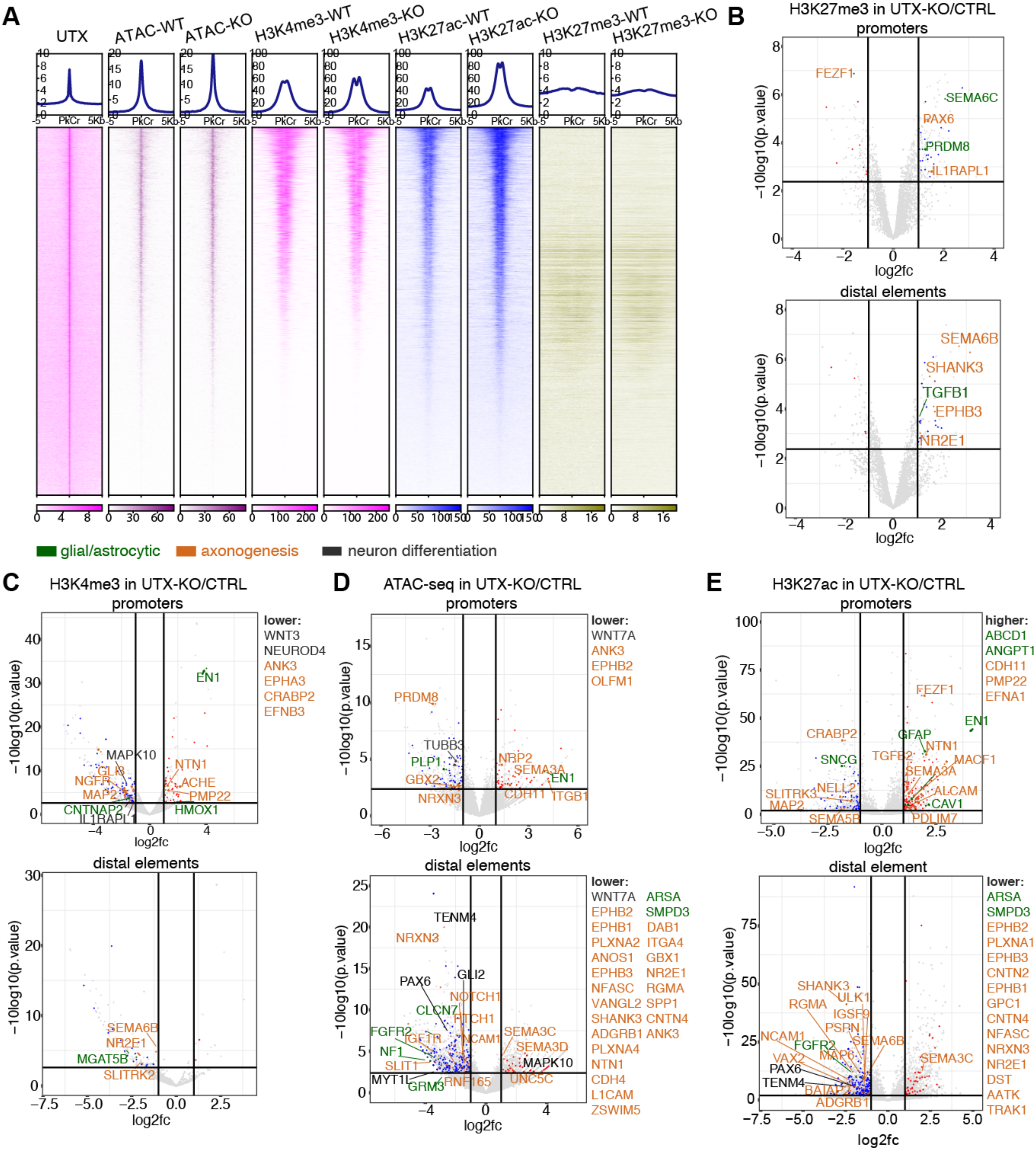

Figure S5. Comparison of chromatin features in UTX-KO cells vs. UTX-WT hNSCs.
(A) Heat map summarizes overlap of UTX-bound regions with the assayed chromatin features. The rows were ordered by signals of all samples using deepTools default with the parameter “plotHeatmap --averageTypeSummaryPlot mean”, with blacklist regions were excluded when generating data matrix by using computeMatrix. Volcano plots of (B) H3K27me3 ChIP-seq, (C) H3K4me3 ChIP-seq, (D) ATAC-seq, and (E) H3K27ac ChIP-seq in the promoters (2kb within transcription start sites) or distal elements (2 to 50kb within

transcription start sites) of UTX KO / control hNSCs. Analysis was done with modified t test in the voom package of R. Notable development-relevant genes for glia/astrocytes, axonogenesis, or neuron differentiation are noted. Lists to the right of perspective volcano plots are of notable genes with significantly lower or higher ChIP- or ATAC-seq levels.

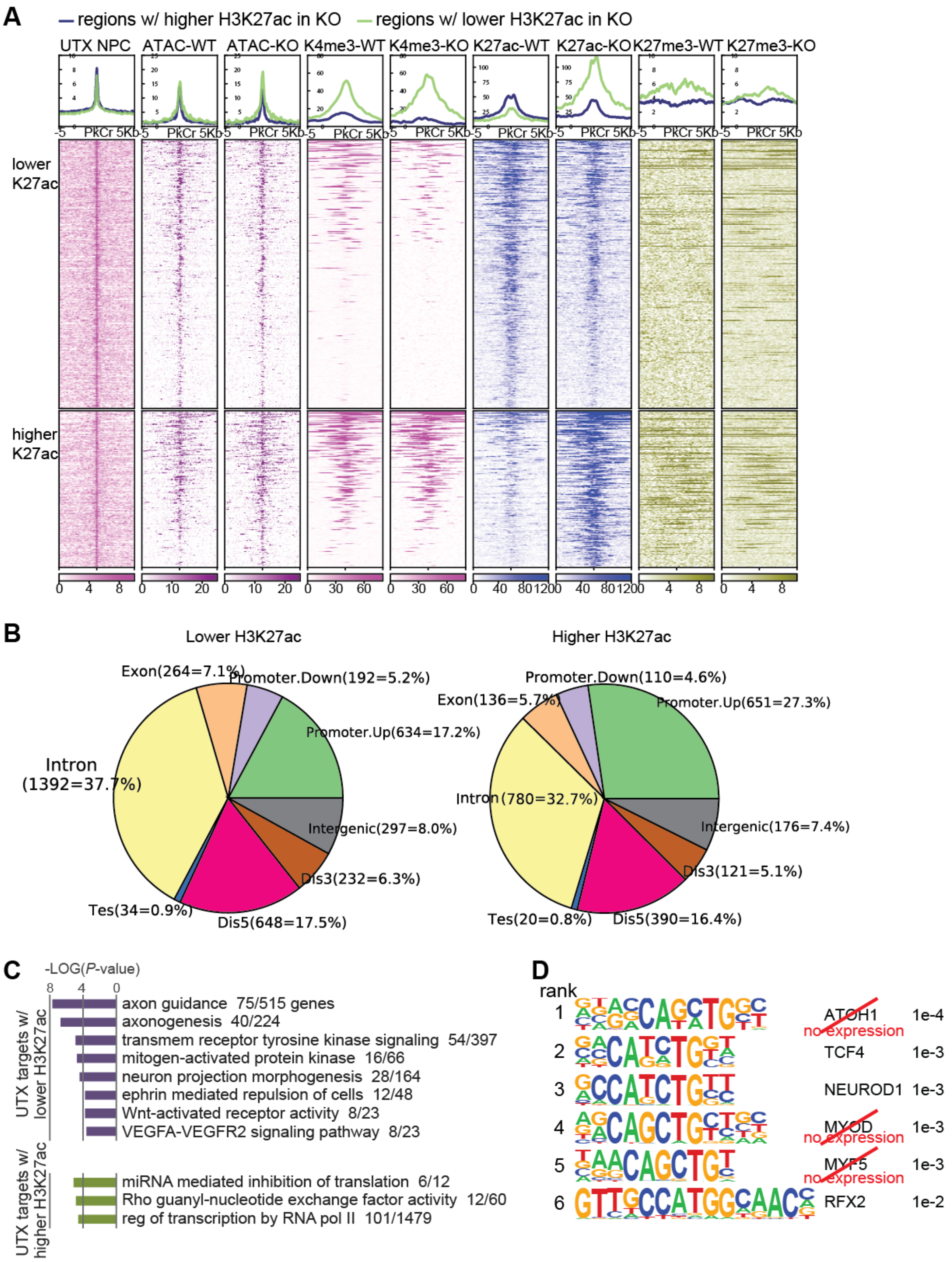
A
regions w/ higher H3K27ac in KO
regions w/ lower H3K27ac in KO
UTX NPC
ATAC-WT
ATAC-KO
K4me3-WT
K4me3-KO
K27ac-WT
K27ac-KO
K27me3-WT
K27me3-KO
-5 PkCr 5Kb
lower K27ac
higher K27ac
B
Lower H3K27ac
Exon(264=7.1%)
Promoter.Down(192=5.2%)
Promoter.Up(634=17.2%)
Intron (1392=37.7%)
Intergenic(297=8.0%)
Dis3(232=6.3%)
Tes(34=0.9%)
Dis5(648=17.5%)
Higher H3K27ac
Promoter.Down(110=4.6%)
Exon(136=5.7%)
Promoter.Up(651=27.3%)
Intron(780=32.7%)
Intergenic(176=7.4%)
Dis3(121=5.1%)
Tes(20=0.8%)
Dis5(390=16.4%)
C
-LOG(P-value)
8 4 0
UTX targets w/ lower H3K27ac
axon guidance 75/515 genes
axonogenesis 40/224
transmem receptor tyrosine kinase signaling 54/397
mitogen-activated protein kinase 16/66
neuron projection morphogenesis 28/164
ephrin mediated repulsion of cells 12/48
Wnt-activated receptor activity 8/23
VEGFA-VEGFR2 signaling pathway 8/23
UTX targets w/ higher H3K27ac
miRNA mediated inhibition of translation 6/12
Rho guanyl-nucleotide exchange factor activity 12/60
reg of transcription by RNA pol II 101/1479
D
rank
1 ATOH1 no expression 1e-4
2 TCF4 1e-3
3 NEUROD1 1e-3
4 MYOD no expression 1e-3
5 MYF5 no expression 1e-3
6 RFX2 1e-2

Figure S6. Comparison of UTX-bound regions that had differential H3K27ac levels in UTX-KO vs. UTX-WT hNSCs.

(A) Heat map summarizes of UTX-bound regions containing differential H3K27ac levels with the assayed chromatin features. (B) Pie charts indicate the proportions of UTX-bound sites with differential H3K27ac levels that are amongst different genic features. ‘Distal’ indicates regions that are 5 kb from transcription start sites. ‘Tes’ denotes transcription termination sites. (C) Gene ontology analysis of UTX-bound genes that had differential H3K27ac levels in UTX-KO vs. UTX-WT hNSCs. Ontology terms were ranked by *P*-value significance, with the number of enriched genes indicated. (D) Ranking, by *P*-values, of motifs enriched in regions with lower H3K27ac signals in UTX-KO hNSCs.

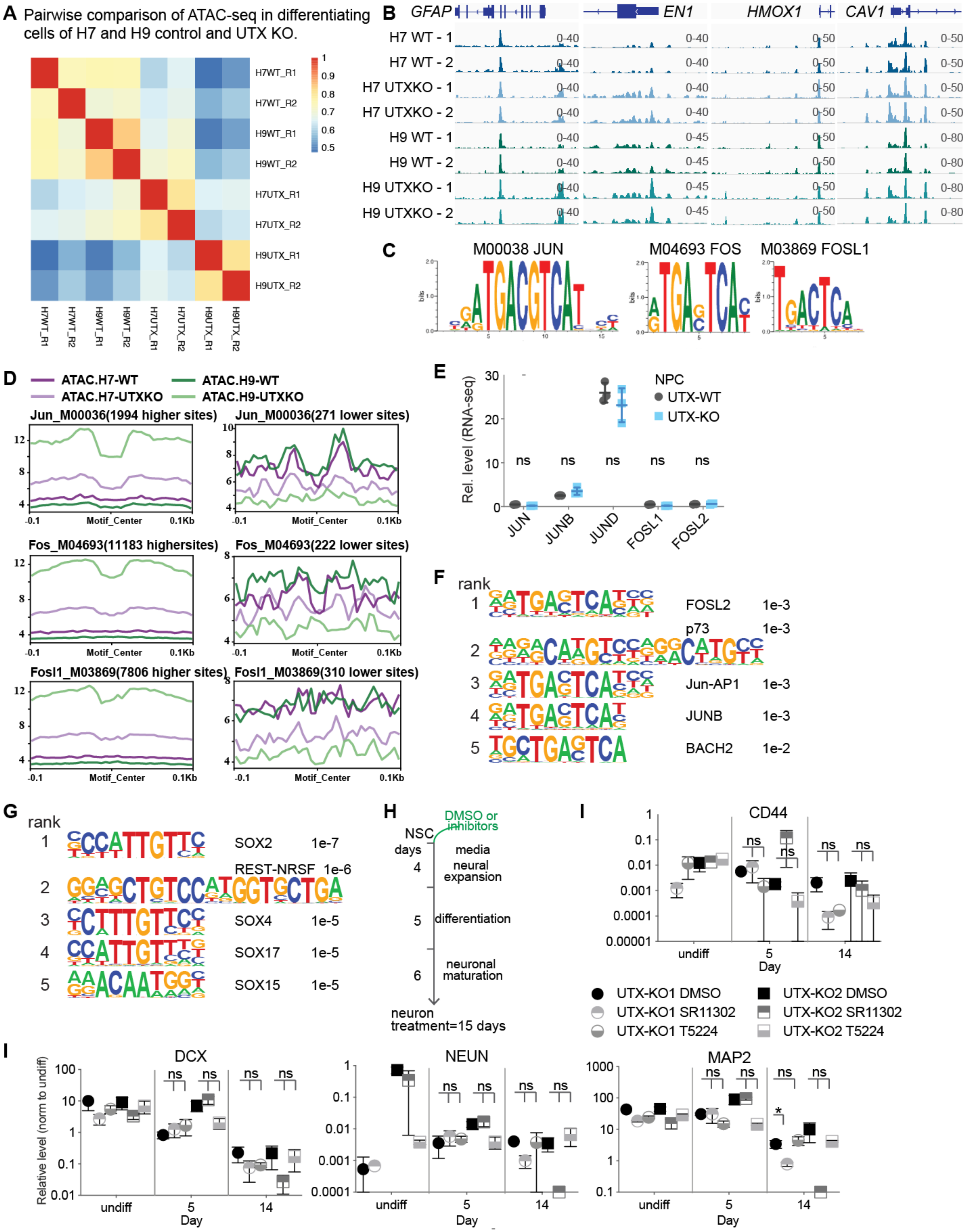
A Pairwise comparison of ATAC-seq in differentiating cells of H7 and H9 control and UTX KO.
H7WT_R1
H7WT_R2
H9WT_R1
H9WT_R2
H7UTX_R1
H7UTX_R2
H9UTX_R1
H9UTX_R2
B GFAP EN1 HMOX1 CAV1
H7 WT - 1
H7 WT - 2
H7 UTXKO - 1
H7 UTXKO - 2
H9 WT - 1
H9 WT - 2
H9 UTXKO - 1
H9 UTXKO - 2
C M00038 JUN M04693 FOS M03869 FOSL1
D ATAC.H7-WT ATAC.H9-WT ATAC.H7-UTXKO ATAC.H9-UTXKO
Jun_M00036(1994 higher sites)
Jun_M00036(271 lower sites)
Fos_M04693(11183 highersites)
Fos_M04693(222 lower sites)
Fosl1_M03869(7806 higher sites)
Fosl1_M03869(310 lower sites)
Motif_Center
E NPC UTX-WT UTX-KO
Rel. level (RNA-seq)
ns
JUN JUNB JUND FOSL1 FOSL2
F rank
1 FOSL2 1e-3
2 p73 1e-3
3 Jun-AP1 1e-3
4 JUNB 1e-3
5 BACH2 1e-2
G rank
1 SOX2 1e-7
2 REST-NRSF 1e-6
3 SOX4 1e-5
4 SOX17 1e-5
5 SOX15 1e-5
H NSC DMSO or inhibitors
days
4 media neural expansion
5 differentiation
6 neuronal maturation
neuron treatment=15 days
I CD44
undiff 5 14 Day
UTX-KO1 DMSO
UTX-KO1 SR11302
UTX-KO1 T5224
UTX-KO2 DMSO
UTX-KO2 SR11302
UTX-KO2 T5224
I DCX NEUN MAP2
Relative level (norm to undiff)

Figure S7. Analyses suggest that UTX suppresses AP-1 subunits.

(A) Pairwise comparisons of datasets of ATAC-seq in control and UTX-KO cells undergoing differentiation. R1 and R2 represents replicates of H7 and H9 UTX-WT or UTX-KO1. (B) ATAC-seq tracks at loci of *GFAP*, *EN1*, *HMOX1*, and *CAV1* in H7 and H9 control and UTX-KO differentiating cells. (C) Consensus motifs of JUN, FOS, and FOSL1. (D) Footprinting analysis of ATAC-Seq profiles surrounding JUN/FOS/FOSL motifs matches("M" number are Motif IDs from TRANSFAC database). Consistently, these sites had increased chromatin accessibility in UTXKO. The lower probability at the motif center Indicated the TFs binding so they were protected from Tn5 insertion. In contrast, this protection were not observed in WTs nor regions with decreased chromatin accessibility. (E) Summary of relative level, normalized by counts per million from RNA-seq datasets, in UTX-WT and UTX-KO hNSCs. Ranking, by *P*-values, of motifs enriched in (F) upregulated and (G) downregulated genes in UTX-KO hNSCs. (H) Schematic diagram of DMSO or AP-1 inhibitor treatment during neuronal differentiation of UTX-KO cells. (I) Analysis of the transcripts of neuronal markers *DCX*, *NEUN*, and *MAP2* and glial/astrocytic marker *CD44* expressed by UTX-KO treated by DMSO or AP-1 inhibitors during neural differentiation. ns indicates not significant. * indicates $P<0.05$ by 1-sided *Student*'s *t* test.

Supplementary Table 1. List of citations supporting UTX-bound genes involved in glial/astrocytic differentiation. Table is in a separate spreadsheet.

Supplementary Table 2. Antibodies used in this study.

**Antibody**	**Species**	**Source**	**Catalogue Number**	**Dilution**	**Validation**
Anti-UTX	Rabbit	Cell Signaling Technology	33510S	WB (1:1000)	PMID: 29227285
Anti-UTX	Rabbit	Millipore	ABE-409	WB (1:1000); IP (6μl)	PMID: 29551674
Anti-UTX	Rabbit	Abcam	ab84190	IP/ChIP (6μl)	PMID: 28974674
Anti-SSEA4	Mouse	STEMCELL Technologies	60062AD	IF (1:200)	PMID: 23728860
Anti-OCT4	Rabbit	Cell Signaling Technology	2840S	IF (1:300)	PMID: 30338036
Anti-MAP2	Chicken	Abcam	ab5392	IF (1:10,000)	PMID: 29348617
Anti-SOX2	Goat	Santa Cruz Biotechnology	sc-17320	IF (1:100)	PMID: 26704449
Anti-OTX2	Goat	R&D Systems	AF1979	IF (1:200)	PMID: 29988106
Anti-SOX1	Rabbit	Cell Signaling Technology	4194S	IF (1:200)	PMID: 29235536
Anti-PAX6	Rabbit	BioLegend	901301	IF (1:200)	PMID: 26879757
Anti-Pericentrin	Rabbit	Abcam	ab4448	IF (1:1000)	PMID: 29133484
Anti-ARL13B	Mouse	NeuroMab	75-287	IF (1:2000)	PMID: 28352462

Anti-Phospho-Histone H3	Rabbit	Millipore Sigma	06-570	IF (1:300)	PMID: 25504633
Anti-BrdU	Mouse	BioLegend	364106	IF (1:100)	PMID: 26265781
Anti-CD44	Mouse	Cell Signaling Technologies	5640S	IF (1:100)	PMID: 28851898
Anti-GFAP	Rabbit	Cell Signaling Technologies	12389S	IF (1:100)	PMID: 29907863
Anti-Ki67	Rabbit	Cell Signaling Technologies	9129S	IF (1:100)	PMID: 29423921
Anti-beta actin	Mouse	Sigma Aldrich	A1978	WB (1:1000)	PMID: 17296734
Anti-beta Tubulin	Mouse	Sigma Aldrich	T8660	IF (1:300)	PMID: 20074521
Anti-Nestin	Mouse	Santa Cruz Biotechnology	sc-23927	IF (1:300)	PMID: 28946814
Anti-CTIP2	Rat	Abcam	ab18465	IF (1:300)	PMID: 29275859
Anti-pS2-RNApol2	Rabbit	Abcam	Ab5095	CUT&RUN (0.5ug)	This study
Anti-Histone H3K27Ac	Rabbit	Abcam	ab4729	ChIP (4µl)	PMID: 30513303
Anti-Histone H3K27me3	Rabbit	Active Motif	39155	ChIP (4µl)	PMID: 30305620
Anti-Histone H3K4me3	Rabbit	Diagenode	C15410003	ChIP (4µl)	PMID: 29078328
Anti-Histone H2Av (Drosophila-specific)	Rabbit	Active Motif	61686	CUT&RUN (0.2ug)	This study
